# Rare-variant collapsing and bioinformatic analyses for different types of cardiac arrhythmias in the UK Biobank reveal novel susceptibility loci and candidate amyloid-forming proteins

**DOI:** 10.1016/j.cvdhj.2023.12.001

**Published:** 2023-12-23

**Authors:** Bengt Zöller, Eric Manderstedt, Christina Lind-Halldén, Christer Halldén

**Affiliations:** ∗ Center for Primary Health Care Research, Department of Clinical Sciences, Lund University and Region Skåne, Malmö, Sweden; † Department of Bioanalysis, Kristianstad University, Kristianstad, Sweden

**Keywords:** Epidemiology, Genetics, Mutation, Arrhythmias, Cardiac

## Abstract

**Background:**

Cardiac arrhythmias are a common health problem. Both common and rare genetic risk factors exist for cardiac arrhythmias. Cardiac amyloidosis is a rare disease that may manifest various arrhythmias. Few large-scale whole exome sequencing studies elucidating the contribution of rare variations to arrhythmias have been published.

**Objective:**

To access gene collapsing analysis of rare variations for different types of cardiac arrhythmias in UK Biobank. Identified genes were analyzed *in silico* for probability to form amyloid fibrils.

**Methods:**

We used 2 published UK Biobank portals (https://azphewas.com/ and https://app.genebass.org/) to access gene collapsing analysis of rare variations for different types of cardiac arrhythmias. Diagnosis of arrhythmia was based on the International Classification of Diseases, 10th Revision (ICD-10) codes: conduction disorders (I44, I45), paroxysmal tachycardia (I47), atrial fibrillation (I48), and other arrhythmias (I49).

**Results:**

Rare variations in 5 genes were linked to conduction disorders (*SCN5A, LMNA*, *SMAD6*, *HSPB9, TMEM95*). The *TTN* gene was associated with both paroxysmal tachycardia and other arrhythmias. Atrial fibrillation was associated with rare variations in 8 genes (*TTN*, *RPL3L, KLF1, TET2, NME3, KDM5B, PKP2, PMVK*). Two of the genes linked to heart conduction disorders were potential amyloid-forming proteins (*HSPB9, TMEM95*), while none of the 8 genes linked to other types of arrhythmias were potential amyloid-forming proteins.

**Conclusion:**

Rare variations in 13 genes were associated with arrhythmias in the UK Biobank. Two of the heart conduction disorder–linked genes are potential amyloid-forming candidates. Amyloid formation may be an underestimated cause of heart conduction disorders.

## Introduction

Cardiac arrhythmias are a common challenge to human health.^1^ The development of effective technologies for the treatment of cardiac arrhythmias has exceeded the knowledge of the underlying biology.^1^ Both acquired and common and rare genetic and risk factors exist for cardiac arrhythmias.^1^^,^^2^ Cardiac amyloidosis is a rare disease that may manifest various arrhythmias, such as atrioventricular nodal block, atrial fibrillation, and ventricular tachyarrhythmias.^2^ Amyloidosis is characterized by extracellular deposits of amyloid in various organs.^2^ Cardiac amyloidosis with cardiomyopathy is a frequent feature of amyloidosis. Patients with cardiac amyloidosis frequently encounter various arrhythmias.^2^ There are 2 main forms of amyloidosis that affect the heart: light chain amyloidosis and transthyretin amyloidosis.^2^ However, there are other rare causes of heart amyloidosis. Genome-wide association studies (GWAS) have been successful in linking common variants to common disease (https://www.ebi.ac.uk/gwas/). Whole exome sequencing studies (WES) might be an interesting option to elucidate the genetics of cardiac arrhythmias. However, few large-scale WES of arrhythmias in the general population have been published.^3^ We have used 2 published UK Biobank portals (https://azphewas.com/ and https://app.genebass.org/) to access gene collapsing analysis of rare variations for different types of cardiac arrhythmias.^4^^,^^5^ Identified genes were further analyzed using bioinformatic sources. The AMYPred-FRL^6^ web server was used to analyze *in silico* whether identified proteins may be potential candidates to form amyloid fibrils.

## Methods

Wang and colleagues^4^ reported the relationships between rare protein-coding variants and 17,361 binary and 1419 quantitative phenotypes using exome sequencing data from 269,171 UK Biobank participants of European ancestry (https://azphewas.com/). Recently, Karczewski and colleagues^5^ determined gene-based associations for 4529 phenotypes in 394,841 UK Biobank exomes (https://app.genebass.org/). The 2 portals used different statistical methods for the analysis.^4^^,^^5^ We used 2 published UK Biobank portals (https://azphewas.com/ and https://app.genebass.org/) to access gene collapsing analysis of rare variations for different types of cardiac arrhythmias (Table 1).^4^^,^^5^ The significance levels used in the 2 portals and published studies were stringent.^4^^,^^5^ In order not to discard potential candidate genes, we present genes with *P* values <.05/20,000 genes = 2.5 × 10^–6^ commonly used for WES studies. In the Astra Zeneca portal the UK Biobank 450k (v4) public version was used (https://azphewas.com/). In Table 1 only the genes with genome-wide significant results are shown with *P* values for the most significant model. Diagnosis of arrhythmia was based on the International Classification of Diseases, 10th Revision (ICD-10) codes (https://azphewas.com/ and https://app.genebass.org/).^4^^,^^5^ Other bioinformatic tools used were the GWAS catalog (https://www.ebi.ac.uk/gwas/), OMIM (https://www.omim.org/), the GeneCards suite (https://www.genecards.org/), and the AMYPred-FRL web server.^6^ The AMYPred-FRL web server was used to analyze *in silico* whether the identified proteins may be potential candidates to form amyloid fibrils (http://pmlabstack.pythonanywhere.com/AMYPred-FRL).^6^ The protein sequences in FASTA format were obtained from UniProt (https://www.uniprot.org/). Validation of the AMYPred software has shown an accuracy of 0.873, sensitivity of 0.848, and specificity of 0.883.^6^

**Table 1:** Results of gene collapsing analysis of rare variants for heart arrhythmia according to 3-digit International Classification of Diseases, 10th Revision codes in 2 published UK Biobank portals (AstraZeneca; https://azphewas.com/ and Genebass; https://app.genebass.org/)^4^^,^^5^

ICD-10 codes and name of diseases	Variant	GWAS catalog	OMIM/GeneCards	AMYPred-FRL amyloid probability^6^
I44 Atrioventricular and left bundle-branch block	*LMNA* 1.03e-6∗ *SMAD6* 1.19e-9^∗^^,^^†^ *HSPB9* 2.13e-6∗ *SCN5A* 2.21e-8†	- - - Brugada syndrome, atrial fibrillation	‡Cardiomyopathy dilated, muscular dystrophy Aortic valve disease Hereditary motor neuropathy (HSPB family) §Atrial fibrillation, heart block	Non-AMY 0.069 non-AMY 0.099 AMY 0.829 Non-AMY 0.090
I45 Other conduction disorders	*SMAD6* 9.80e-9∗^,^^†^ *TMEM95* 1.63e-6^†^	- -	Aortic valve disease -	Non-AMY 0.099 AMY 0.692
I47 Paroxysmal tachycardia	*TTN* 2.84e-9∗	Atrial fibrillation, heart failure	Cardiomyopathy, muscular dystrophy, myopathy	Non-AMY 0.075
I48 Atrial fibrillation and flutter	*TTN* 8.05e-24∗ *RPL3L* 9.55e-13∗^,^^†^ *KLF1* 2.56e-8∗ *TET2* 2.85e-8∗ *NME3* 2.86e-7∗^,^^†^ *KDM5B* 3.13e-12^†^ *PKP2* 1.62e-6^†^ *PMVK* 1.37e-7^†^	Atrial fibrillation, heart failure Atrial fibrillation - Prostate carcinoma, COPD - Mitral valve prolapse Atrial fibrillation Atrial fibrillation, Parkinson disease	Cardiomyopathy, muscular dystrophy, myopathy Cardiomyopathy Dyserythropoietic anemia, blood group Myelodysplastic syndrome, immunodeficiency Lipase deficiency, Heinz Body anemias Intellectual developmental disorder, autism Arrhythmogenic dysplasia and cardiomyopathy Porokeratosis	Non-AMY 0.075 Non-AMY 0.053 Non-AMY 0.252 Non-AMY 0.183 Non-AMY 0.134 Non-AMY 0.072 Non-AMY 0.073 Non-AMY 0.278
I49 Other cardiac arrhythmias	*TTN* 6.14e-11∗	Atrial fibrillation, heart failure	Cardiomyopathy, muscular dystrophy, myopathy	Non-AMY 0.075

COPD = chronic obstructive pulmonary disease; ICD-10 = International Classification of Diseases, 10th Revision.

Union was used to define phenotypes for https://azphewas.com. Only genome-wide significantly associated genes are shown in the table (best model), ie, *P* < 2.5 × 10^-6^ (^∗^significant in AstraZeneca portal [https://azphewas.com]; ^†^significant in Genebass portal [https://app.genebass.org]. In the Astra Zeneca portal the UK Biobank 450k (v4) public version was used (https://azphewas.com/). Three genes were genome-wide significant in the 2 databases, the others only in AstraZeneca portal or Genebass portal.^4^^,^^5^

Significance threshold was *P* value <.05/20,000 genes = 2.5 × 10^–6^, commonly used for WES studies. For the Genebass portal (https://app.genebass.org) the highest value for SKAT-O, SKAT, or burden test are shown. For the AstraZeneca portal (https://azphewas.com) the highest *P* value of the 12 tested models is shown.^4^^,^^5^

Other bioinformatic tools used were the GWAS catalog (https://www.ebi.ac.uk/gwas/), OMIM (https://www.omim.org/), the GeneCards suite (https://www.genecards.org/), and the AMYPred-FRL web server.^6^ AMYPred-FRL results were obtained from the http://pmlabstack.pythonanywhere.com/AMYPred-FRL^6^ server using the respective protein sequence in FASTA format obtained from https://www.uniprot.org/.

‡ Associated also with Hutchinson-Gilford progeria, Charcot-Marie-Tooth disease, Emery-Dreifuss muscular dystrophy, lipodystrophy, Malouf syndrome, restrictive dermopathy, mandibuloacral dysplasia, heart-hand syndrome.

§ Associated also with Brugada syndrome, cardiomyopathy, long QT syndrome, sick sinus syndrome, ventricular fibrillation.

## Results

One previously GWAS-linked gene (*SCN5A*) and 3 novel genes (*LMNA*, *SMAD6*, *HSPB9*) were identified for atrioventricular and left bundle branch block (ICD10 code I44). Only loss-of-function variants in the *SCN5A* were associated with I44. For other conduction disorders several novel associations were identified. The *SMAD6* and *TMEM95* genes were associated with ICD10 code I45. One gene (*TTN*) was associated with both paroxysmal tachycardia (ICD10 code I47) and other cardiac arrhythmias (ICD10 code I49). Atrial fibrillation (ICD10 code I48) was associated with rare variation in 8 genes (*TTN*, *RPL3L, KLF1, TET2, NME3, KDM5B, PKP2, PMVK*). Four of these genes have previously been linked in GWAS to atrial fibrillation (*TTN, RPL3L, PKP2, PMVK*).

Using AMYPred-FRL the *HSPB9* and *TMEM95* proteins were predicted to be novel amyloid-forming candidates with probabilities of 0.829 and 0.692, respectively. The other 11 proteins had low *in silico* probability to form amyloid fibrils (Table 1). Thus, 2 of the genes linked to heart conduction disorders were potential amyloid-forming proteins, while none of the 8 genes linked to other arrhythmias were potential amyloid-forming proteins.

## Discussion

The present study shows that rare variations in 13 genes are associated with different types of arrhythmias in UK Biobank. Eight of these associations were novel (*LMNA*, *SMAD6*, *HSPB9, TMEM95, KLF1, TET2, NME3, KDM5B*) and 5 genes have previously been linked to arrhythmias (*SCN5A, TTN, RPL3L, PKP2, PMVK*). Of special interest is that 2 of the genes linked to heart conduction disorders were potential amyloid-forming proteins (*HSPB9, TMEM95)*, while none of the 8 genes linked to other arrhythmias were potential amyloid-forming proteins.

Previously genes coding for heat shock protein family B (*HSPB1, HSPB3, HSPB5, HSPB8*) closely related to the *HSPB9* gene have been associated with distal hereditary motor neuropathy (https://www.genecards.org/).^7^ Neuropathy is a known manifestation of amyloidosis (https://www.omim.org/), which further strengthens our finding that *HSPB9* codes for a potential amyloid-forming protein. No association between the *TMEM95* gene and amyloid or arrhythmia has been reported. Another interesting finding is the link between atrial fibrillation and rare variations in the *TET2* gene. The *TET2* gene is one of the driver genes involved in clonal hematopoiesis of indeterminate potential (CHIP) and has been linked to cardiovascular diseases (https://www.omim.org/). Clonal hematopoiesis of indeterminate potential involves proinflammatory macrophages and an inflammasome-dependent immune response (interleukin-1 and interleukin-6) in atherosclerotic plaques or directly in the myocardium.^8^ Myocardial inflammation may induce cardiac fibrosis, even in the absence of atherosclerotic cardiovascular disease.^8^

### Strengths and limitations

A strength of our study is the large number of sequenced exomes in UK Biobank.^4^^,^^5^ A limitation is the lack of validation studies of our findings. Other limitations are the lack of experimental evidence and the use of 3-digit ICD-10 codes for the diagnosis of cardiac arrhythmias in UK Biobank. Certain ICD-10 codes are heterogeneous in terms of electrophysiological mechanisms (ie, I44, I45, I47, I49). Moreover, owing to the large number of comparisons some associations might be spurious, although we applied the genome-wide threshold 2.5 × 10^–6^ commonly used for WES studies.

## Conclusion

In conclusion, rare variations in 13 genes were associated with different types of arrhythmias in UK Biobank. Two of the heart conduction disorders linked genes are potential amyloid-forming candidates. We therefore hypothesize that amyloid formation could be an underestimated cause of heart conduction disorders.

## References

[1] Grace A.A., Roden D.M. (2012). Systems biology and cardiac arrhythmias. Lancet.

[2] Laptseva N., Rossi V.A., Sudano I. (2023). Arrhythmic manifestations of cardiac amyloidosis: challenges in risk stratification and clinical management. J Clin Med.

[3] Kandola M.S., Kulm S., Kim L.K. (2023). Population-level prevalence of rare variants associated with atrial fibrillation and its impact on patient outcomes. JACC Clin Electrophysiol.

[4] Wang Q., Dhindsa R.S., Carss K. (2021). Rare variant contribution to human disease in 281,104 UK Biobank exomes. Nature.

[5] Karczewski K.J., Solomonson M., Chao K.R. (2022). Systematic single-variant and gene-based association testing of thousands of phenotypes in 394,841 UK Biobank exomes. Cell Genom.

[6] Charoenkwan P., Ahmed S., Nantasenamat C. (2022). AMYPred-FRL is a novel approach for accurate prediction of amyloid proteins by using feature representation learning. Sci Rep.

[7] Sarparanta J., Jonson P.H., Kawan S., Udd B. (2020). Neuromuscular diseases due to chaperone mutations: a review and some new results. Int J Mol Sci.

[8] Sikking M.A., Stroeks S.L.V.M., Waring O.J. (2023). Clonal hematopoiesis of indeterminate potential from a heart failure specialist’s point of view. J Am Heart Assoc.

